# Fatty Liver Is Associated with Low N-Terminal Pro-B-Type Natriuretic Peptide in a Healthy Population

**DOI:** 10.3390/jcm10071402

**Published:** 2021-04-01

**Authors:** Hyo-In Choi, Mi Yeon Lee, Byeong Kil Oh, Seung Jae Lee, Jeong Gyu Kang, Sung Ho Lee, Jong-Young Lee, Byung Jin Kim, Bum Soo Kim, Jin Ho Kang, Ki-Chul Sung

**Affiliations:** 1 Division of Cardiology, Department of Internal Medicine, Kangbuk Samsung Hospital, Sungkyunkwan University School of Medicine, Seoul 03181, Korea; drhyoin.choi@samsung.com (H.-I.C.); bkil.oh@samsung.com (B.K.O.); sj0519.lee@samsung.com (S.J.L.); shsh96.lee@samsung.com (S.H.L.); jyleeheart.lee@samsung.com (J.-Y.L.); bjjake.kim@samsung.com (B.J.K.); bsmed.kim@samsung.com (B.S.K.); jinho2.kang@samsung.com (J.H.K.); 2 Division of Biostatistics, Department of R&D Management, Kangbuk Samsung Hospital, Sungkyunkwan University School of Medicine, Seoul 03181, Korea; my7713.lee@samsung.com; 3 Center for Cohort Studies, Total Healthcare Center, Kangbuk Samsung Hospital, Sungkyunkwan University School of Medicine, Seoul 03181, Korea; jg1980.kang@samsung.com

**Keywords:** biomarkers, insulin resistance, epidemiology, liver/diagnostic imaging

## Abstract

Fatty liver (FL), insulin resistance (IR) and obesity often coexist, but data on the independent impacts of these factors on N-terminal pro-B-type natriuretic peptide (NT-proBNP) levels are scarce. We examined the impact of FL, IR and obesity on NT-proBNP levels using a large set of cross-sectional data. The associations of FL, IR and obesity with NT-proBNP were analyzed in 39,923 healthy adult participants. IR was estimated using a homeostasis model assessment-estimated insulin resistance (HOMA-IR) index. A multivariable regression model was conducted to identify associations between NT-proBNP and FL on abdominal ultrasound. FL, IR and obesity showed independent inverse associations with NT-proBNP after multiple adjustments for baseline characteristics. In a multivariable regression model adjusting for IR and obesity, FL was independently associated with lower levels of NT-proBNP (estimates, Exp(β) 0.864, 0.849–0.880). The combination of FL and IR was a powerful dual indicator, lowering NT-proBNP levels approximately 25% in the generally healthy study population. In conclusion, FL was independently associated with lower NT-proBNP levels. FL and a high HOMA-IR index are a powerful indicator combination for lower NT-proBNP levels. Further research is needed to elucidate the mechanism underlying the association between FL and NT-proBNP.

## 1. Introduction

Levels of B-type natriuretic peptide (BNP) and N-terminal proBNP (NT-proBNP) help to distinguish cardiac causes of dyspnea, and are also useful in the prediction of prognosis and monitoring the treatment efficacy in patients with heart failure [1]. In particular, the addition of NT-proBNP measurements to traditional risk assessment in individuals without cardiovascular disease (CVD) can increase the accuracy of the prediction of future CVD or cardiovascular events.

Novel metabolic functions of NPs, including lipolysis activation, lipid oxidation and mitochondrial respiration [2], have been identified in recent years. NPs can also ameliorate lipid-induced insulin resistance (IR) through improvements in hepatic [3] and muscular [4] lipid oxidation. These actions combine to enhance the browning and oxidative potential of white adipose tissue and protect individuals against obesity and IR. Additionally, some data suggest that circulating glucose and insulin alter NP levels. In one study of obese subjects, hyperinsulinemia significantly reduced levels of NP by increasing the expression of the NP-clearance receptor in subcutaneous fat tissue [5]. Based on previous observations, hyperglycemia and hyperinsulinemia may be the mechanisms underlying NP deficiency in obesity and diabetes.

Fatty liver (FL) is common in patients with obesity or IR. Both obesity and IR are associated with lower levels of NT-proBNP. Some studies have demonstrated that FL is associated with low NT-proBNP [6,7,8]. Whether an IR or obesity-specific factor decreases NT-proBNP levels in FL populations or whether common pathological mechanisms exist in FL and IR/obesity, which decrease NT-proBNP levels is currently uncertain. The availability of routine plasma NT-proBNP measurements and abdominal ultrasound (US) on >30,000 young individuals in the Kangbuk Samsung Health Study allows for a comprehensive investigation of the association of these variables and NT-proBNP levels. Therefore, we investigated the associations between FL, IR and obesity and NT-proBNP levels in a large, relatively young, healthy and well-characterized occupational cohort.

## 2. Materials and Methods

### 2.1. Study Population

This study included 44,066 individuals who were enrolled in the Kangbuk Samsung Health Study (KSHS) from 2016 to 2018 and had their NT-proBNP measurements taken and abdominal US. The KSHS is a retrospective cohort study that has been ongoing for those aged 18 or older who underwent a comprehensive examination at the Total Healthcare Centers of the Kangbuk Samsung Hospital since 2002. Over 80% of participants in the KSHS were employees of various companies or local government organizations. The remaining 20% of participants voluntarily applied for self-paid screening examinations at the health screening center. The screening program aimed to promote health through early detection of chronic diseases and their risk factors. Additionally, the Korean Industrial Safety and Health Law requires employees to participate in an annual or biennial health examination. To minimize the effect of disease or medication on NT-proBNP levels, we excluded 4143 subjects due to hypertension (*n* = 2180), coronary disease (*n* = 140), diabetes (*n* = 821), history of cancer (*n* = 1285), NT-proBNP > 18,000 (*n* = 3) and missing information (*n* = 193). Those taking heart-related medications were also excluded, and some participants met multiple conditions of exclusion; 39,923 participants were included in the final study. This study was approved by the Institutional Review Board of Kangbuk Samsung Hospital (Reference number: KBSMC 2018-06-001), which waived the requirement for informed consent because only retrospectively accessed de-identified data were utilized.

### 2.2. Measurements

Data on demographic variables, health behaviors, educational background, past medical history and family history of CVD were collected using standardized, self-administered questionnaires [9]. Anthropometric measurements and vital statistics were obtained by professional staff. The questionnaire asked about the frequency of alcohol consumption and the amount of alcohol consumed per consumption day, recorded in standard units [10]. Body weight was measured in light clothing with no shoes to the nearest 0.1 kg using a digital scale. Height was measured to the nearest 0.1 cm. Body mass index (BMI) was calculated as weight in kilograms divided by height in meters squared.

Physical activity level was assessed using the validated Korean version of the International Physical Activity Questionnaire Short Form [11] and categorized as sedentary, mild, or health-enhancing physical activity (HEPA) [12]. HEPA was categorized if either of the following criteria was met: (i) vigorous intensity activity on ≥3 days per week and accumulating ≥1500 metabolic equivalents minute (MET)/week or (ii) 7 days of any combination of walking, moderate intensity, or vigorous intensity activities, achieving at least 3000 MET minute/week. Physical activity was characterized as mild if any of the following criteria were met: (i) ≥3 days of vigorous activity for ≥20 min/day, (ii) ≥5 days of moderate intensity activity or walking for ≥30 min/day, or (iii) ≥5 days of any combination of walking and moderate or vigorous intensity activities achieving ≥600 MET minute/week. Physical activity was characterized as sedentary if it was not characterized as either mild or HEPA.

Following a minimum of 10 h of fasting, blood samples were obtained and analyzed in a single clinical core laboratory. The core clinical laboratory is certified by the Korean Association of Quality Assurance for Clinical Laboratories. Serum levels of total cholesterol, triglycerides, low-density lipoprotein cholesterol (LDL-C) and high-density lipoprotein cholesterol (HDL-C) were measured using Bayer Reagent Packs (Bayer Diagnostics, Leverkusen, Germany) on an automated chemistry analyzer (Advia 1650 Autoanalyzer; Bayer Diagnostics). Serum creatinine level was measured using the kinetic alkaline picrate method (Jaffe method) on an automated chemistry analyzer (Modular D2400 [Roche]). The eGFR was calculated using the CKD-EPI equation as described by Levey et al. [13,14]. eGFR was expressed in milliliters per minute per 1.73 m^2^. Serum NT-proBNP levels were determined using an automated immunoassay analyzer (cobas e411; Roche Diagnostics, Tokyo, Japan). The inter-assay coefficients of variation for quality control specimens of lower levels and higher levels were 1.20–5.37% and 2.28–4.33%, respectively, during the study period. 

The diagnosis of FL was based on abdominal US performed by experienced radiologists who were blinded to the aim of the present study. Ultrasonographic diagnosis of FL was determined based on standard criteria, including a diffuse increase of fine echoes in the liver parenchyma compared with kidney or spleen parenchyma, deep beam attenuation and bright vessel walls [15]. The HOMA-IR was calculated as fasting insulin (mg/dL) × fasting glucose (mg/dL)/405. Individuals with HOMA-IR in the top quartile were considered to have IR [16]. Obesity was defined as BMI ≥25 kg/m^2^ in this Asian population.

### 2.3. Statistical Analysis

Data are expressed as means (standard deviation (SD)) or as median (interquartile ranges) for continuous variables. Categorical data are expressed as numbers and percentages. Baseline variables were compared using Student’s t-test, Mann-Whitney U test or chi-square test as appropriate. The distribution of each variable was verified using a graphical method (histograms). NT-proBNP showed a right-skewed distribution and was transformed into log_2_ to facilitate interpretation. Regression analyses were repeated using generalized linear models to accommodate variables (FL, HOMA-IR index and BMI) with log_2_-transformed NT-proBNP, using non-FL and low HOMA-IR index as the reference. Estimated value means the exponential coefficient (exp(β)) from the generalized linear model. Multivariate model 1 was adjusted for age, sex, waist circumference, systolic blood pressure, smoking status, physical activity, alcohol consumption, educational level and estimated glomerular filtration rate. To assess whether the association between FL and NT-proBNP level is mediated by IR, model 2 was adjusted for the variables in model 1, and BMI and HOMA-IR index. Reported *p* values were two-tailed, and <0.05 was considered statistically significant. All statistical analyses were conducted using STATA version 16.1 (StataCorp LP, College Station, TX, USA).

## 3. Results

A total of 39,923 individuals were included in the final analysis. The mean age of the population was 39.1 and 57.6% were male, and 11,704 (29.3%) individuals had FL according to their abdominal US. Baseline characteristics according to the presence of FL are described in Table 1. Individuals with FL were older, were more likely to be obese, consumed more alcohol and had higher LDL-cholesterol levels than the non-FL group. The non-FL group was associated with health-enhancing physical activity (HEPA) and better renal function. Liver enzymes were slightly higher in the FL group. The FL group showed a higher median HOMA-IR index (2.0 and 1.2 for the FL group and non-FL group, respectively). NT-proBNP level was significantly lower in the FL group (14.5 ng/dL for the FL group vs. 24.4 ng/dL for the non-fatty liver group). 

To assess the association between IR and NT-proBNP levels, we divided the overall population into two groups according to quartiles of the HOMA-IR index. Individuals with HOMA-IR in the top quartile were considered to have IR; 9980 (25.0%) of individuals were categorized as having IR (Table 2). Individuals without IR were older than individuals with IR. Individuals with IR had higher liver enzyme levels, BMI, and blood cholesterol levels. NT-proBNP was significantly lower in the IR group (22.8 for the non-IR group vs. 15.5 for the IR group). Nearly 60% of individuals in the IR group had FL (19.7% for the low HOMA-IR group vs. 58.2% for the high HOMA-IR group).

When stratified by sex (Table 3), males were older, more likely to be obese, consumed more alcohol, and had higher blood pressure, glucose, and cholesterol levels than females. Males showed a higher HOMA-IR index (1.5 for males vs. 1.3 for females) and had FL more frequently (43.0% for males vs. 10.6% for females) compared to females. NT-proBNP level was significantly higher in females than males (14.6 vs. 32.8 for males and females, respectively).

We evaluated the effect of each variable (FL, HOMA-IR index and BMI) on NT-proBNP levels using multivariable regression analysis (Table 4). Models were adjusted for sex, age, waist circumference, systolic blood pressure, smoking status, alcohol intake, educational level and estimated glomerular filtration rate. In a multivariable model (model 1), FL was associated with lower levels of NT-proBNP in the overall population (estimate 0.83, 0.82–0.85), and this inverse association was weaker in males. Similarly, higher HOMA-IR index was associated with lower NT-proBNP levels after multivariable adjustment (estimate 0.86, 0.84–0.87). Obesity (as defined by BMI ≥ 25) was also associated with lower NT-proBNP levels (estimate 0.95, 0.93–0.97). These trends for inverse association were observed in both sexes with identical patterns (weaker in males, *p* for trend by sex < 0.001). We also made a multivariable regression model that adjusted for HOMA-IR score and BMI (model 2) to assess the independent effect of FL on NT-proBNP level; FL still showed an inverse association with NT-proBNP level (estimate 0.86, 0.85–0.88) in this model.

**Figure 1:**
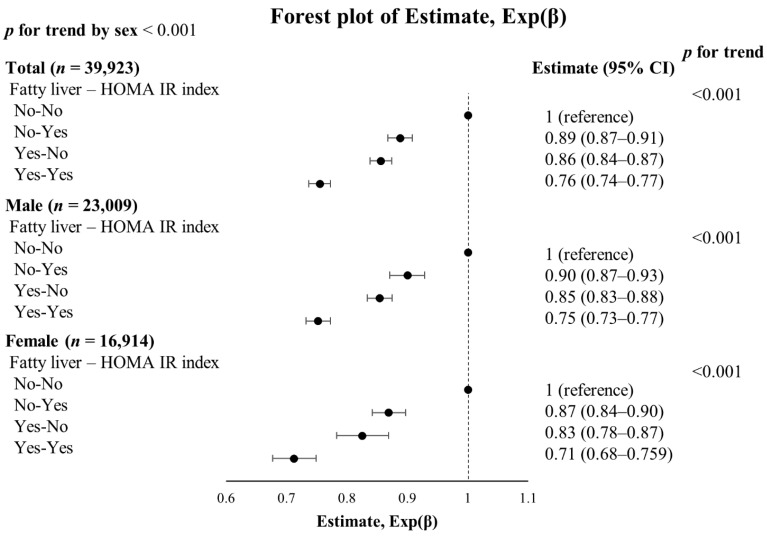
Forest plots are shown depicting the estimates, Exp(β) for N-terminal pro-B-natriuretic peptide (NT-proBNP) levels in a multivariate logistic regression analysis. Fatty liver and HOMA-IR index (top quartile) were treated as categorical variables. Data points represent the OR ± the standard error. 95% CI indicates 95% confidence interval.

We determined whether or not the presence of FL or high HOMA-IR score affects NT-proBNP levels (Table 5, Figure 1). Using NT-proBNP of the group both without FL and having a low HOMA-IR score as reference, high HOMA-IR scores and FL were associated with low levels of NT-proBNP. Individuals with both FL and high HOMA-IR index were associated with 25% reduction in NT-proBNP levels as compared to NT-proBNP levels in individuals without both FL and IR. 

## 4. Discussion

The novel finding of our study was that FL is an independent indicator for lower NT-proBNP levels in a generally healthy population. After adjustment for baseline characteristics, FL, HOMA-IR index, and BMI were inversely associated with NT-proBNP level. FL had an independent association with NT-proBNP level in a multivariable regression model after adjustment for HOMA-IR index and BMI. Also, our data showed that the combination of FL and high HOMA-IR index is a powerful indicator combination, lowering NT-proBNP levels approximately 25% in our generally healthy population.

IR is a risk factor for diabetes or CVD due to its association with metabolic syndrome; this was demonstrated by Reaven et al. in 1988. The euglycemic clamping test is accurate and continues to be the gold standard procedure for measuring IR [17], but its complexity limits its application in daily medical practice. IR assessment using HOMA-IR has been the most frequently used technique, both in clinical practice and in epidemiological studies due to the simplicity of its determination and measurement. Obesity and FL can be used as other indicators of IR.

FL can easily be evaluated by abdominal US; and FL’s association with metabolic syndrome, diabetes and CVD has been demonstrated, making the presence of FL a good surrogate marker. Also, FL is closely linked to hepatic IR as the HOMA-IR index measures peripheral IR. One report provided evidence to support the causal relationship between hepatic fat accumulation and hepatic IR. This research group showed a dose response relationship between hepatic fat accumulation and hepatic IR, and preventing hepatic fat accumulation abrogated the development of hepatic IR [18].

Using a multivariable regression model adjusted for the HOMA-IR index, which is the most representative indicator of IR, we found that FL is associated with low NT-proBNP levels. An especially interesting finding of our study was that when considering FL and HOMA-IR index together, these variables had synergistic effects on low NT-proBNP levels. The previous Dallas Heart Study supports our results. That study demonstrated an inverse association between BNP/NT-proBNP and liver fat using 1H-MR spectroscopy [19]. In our study, this association remained significant after adjusting for BMI and HOMA-IR score, indicating that liver fat is associated with NT-proBNP levels regardless of obesity or IR. In addition, several other researchers have shown that NT-proBNP levels in individuals with magnetic resonance imaging- or computed tomography-defined non-alcoholic fatty liver disease were decreased significantly [7,8]. These findings are supported by the evidence that NT-proBNP levels are affected by circulating glucose or insulin levels. Hyperinsulinemia has been shown to decrease NP-binding receptors and increase clearance receptors in adipose tissues, leading to lower circulating NP levels, while fasting causes the opposite effect [20]. Since there is an abundance of data suggesting that increased NP levels are protective against IR [19], type 2 diabetes mellitus [21,22] and CVD [23,24], lower levels of NT-proBNP may have opposite effects on IR, diabetes and CVD. Besides the epidemiologic evidence, binding of NP to its receptors stimulates an increase in mitochondrial density, oxygen consumption and insulin sensitivity [25,26], which leads to increased lipolysis in adipose tissue [27,28]. The biological explanation for the accumulation of fat associated with low NT-proBNP can be provided by these metabolic actions. However, this is a cross-sectional study, and it is difficult to determine the causal relationship, so it should be regarded as hypothesis-generating, which may then be developed into testable questions. Further large studies and experimental research are needed to confirm their relationship. These results indicate that higher levels of NT-proBNP may exert protective effects against many cardiovascular risk factors, and NT-proBNP may be a missing link between FL and increased risks of cardiovascular outcomes. Since FL can be easily diagnosed with US, identification of FL provides a potentially useful strategy for finding subjects at increased risk of diabetes or CVD in a healthy population.

There are strengths and limitations in our study, which should be considered when interpreting our findings. We have excluded factors that can influence NT-proBNP to assess the precise association between IR and NT-proBNP, and this is one of the strengths of our research. The large number of individuals included, even after individuals with these factors were excluded, is another strength of our study. Due to the cross-sectional design of our study, our results cannot be interpreted as a causal relationship and should be considered as hypothesis-generating. The presence of FL was assessed using abdominal US, but the sensitivity of US for detecting FL is limited to identification of > 25% fat infiltration [29]. We have also used HOMA-IR > 75% as a marker of IR because more sensitive or specific measurements of insulin sensitivity were not available in this cohort. Since KSHS includes relatively young individuals, there may be limitations in directly applying our results to elderly people. Further comprehensive study is needed to determine the underlying mechanism of lower NT-proBNP in subjects with FL.

## 5. Conclusions

In this large sample of healthy individuals, FL was independently associated with lower NT-proBNP levels. FL and a high HOMA-IR index remain a powerful indicator combination for lower NT-proBNP levels. Further research is needed to elucidate the mechanism underlying the association between FL and NT-proBNP.

## Figures and Tables

**Table 1:** Baseline characteristics according to fatty liver on abdominal ultrasound.

	Total (*n* = 39,923)	Fatty Liver (*n* = 11,704)	No Fatty Liver (*n* = 28,219)	*p* Value
Age	39.1 ± 7.6	40.6 ± 7.5	38.4 ± 7.6	<0.001
IPAQ %				<0.001
Sedentary	19,493 (48.83)	5602 (47.86)	13,891 (49.23)	
Mild	14,288 (35.79)	4500 (38.45)	9788 (34.69)	
HEPA	5998 (15.03)	1559 (13.32)	4439 (15.73)	
Unknown	141 (0.35)	43 (0.37)	98 (0.35)	
Current smoker, %	4814 (12.06)	2164 (18.49)	2650 (9.39)	<0.001
Alcohol intake (g/day)	4 (1–13)	6 (2–15)	4 (1–11)	<0.001
High alcohol intake, %	3964 (10.58)	1394 (12.48)	2570 (9.78)	<0.001
BMI, kg/m^2^	23.4 ± 3.4	26.3 ± 3.1	22.3 ± 2.7	<0.001
Waist, cm	81.1 ± 9.8	89.6 ± 8	77.6 ± 8.3	<0.001
Weight, kg	67 ± 13.3	77.6 ± 11.7	62.6 ± 11.3	<0.001
Higher education, %	33,449 (83.79)	9905 (84.63)	23,544 (83.44)	0.012
SBP, mmHg	109 ± 12.1	115.4 ± 11.4	106.4 ± 11.3	<0.001
DBP, mmHg	69.9 ± 9.4	74.6 ± 9.1	67.9 ± 8.8	<0.001
Fasting glucose, mg/dL	93.7 ± 10.9	98 ± 13.9	92 ± 8.7	<0.001
Total cholesterol, mg/dL	190.5 ± 33.1	201.6 ± 34.6	185.9 ± 31.2	<0.001
LDL-C, mg/dL	127.1 ± 32.1	140.9 ± 32.3	121.4 ± 30.3	<0.001
HDL-C, mg/dL	61.2 ± 16.6	50.4 ± 12.2	65.7 ± 16.1	<0.001
Triglycerides, mg/dL	91 (65–135)	140 (101–197)	78 (59–108)	<0.001
AST, IU/L	20 (16–24)	23 (19–30)	19 (16–22)	<0.001
ALT, IU/L	19 (13–28)	30 (21–45)	16 (12–22)	<0.001
rGTP, IU/L	20 (14–34)	34 (23–54)	17 (12–25)	<0.001
NT-proBNP, ng/L	20.76 (12.79–35.8)	14.5 (8.99–23.5)	24.4 (14.5–40.4)	<0.001
eGFR, ml/min/1.73 m^2^	104.1 ± 13.2	101.1 ± 13	105.3 ± 13	<0.001
HOMA-IR	1.38 (0.93–2.04)	2.03 (1.41–2.92)	1.19 (0.82–1.7)	<0.001

Numbers in the table are mean (standard deviation), median (interquartile range), or percentages. ALT = alanine aminotransferase; AST = aspartate aminotransferase; BMI = body mass index; DBP = diastolic blood pressure; eGFR = estimated glomerular filtration rate; HDL-C = high-density lipoprotein cholesterol; HEPA = health enhancing physical activity; HOMA-IR = homeostasis model assessment-estimated insulin resistance; IPAQ = international physical activity questionnaire; LDL-C = low-density lipoprotein cholesterol; NT-proBNP = N-terminal pro-B-type natriuretic peptide; rGTP = γ-glutamyl transpeptidase; SBP = systolic blood pressure. High alcohol intake defined as >30 g/day for men and >20 g/day for women; higher education defined as college graduate or higher.

**Table 2:** Baseline characteristics according to HOMA-IR index.

	Total (*n* = 39,923)	HOMA IR < 75% (*n* = 29,943)	HOMA IR ≥ 75% (*n* = 9980)	*p* Value
HOMA-IR, range	0.04–30.93	0.04–2.03	2.03–30.93	
Age	39.1 ± 7.6	39.1 ± 7.6	38.8 ± 7.6	<0.001
IPAQ %				<0.001
sedentary	19,501 (48.83)	14,289 (47.71)	5212 (52.2)	
Mild	14,293 (35.79)	10,725 (35.81)	3568 (35.74)	
HEPA	6001 (15.03)	4835 (16.14)	1166 (11.68)	
Unknown	141 (0.35)	103 (0.34)	38 (0.38)	
Current smoker, %	4817 (12.06)	3387 (11.31)	1430 (14.32)	<0.001
Alcohol intake (g/day)	4 (1–13)	4 (1–11)	5 (1–14)	<0.001
High alcohol intake, %	3967 (10.59)	2888 (10.29)	1079 (11.48)	0.001
BMI, kg/m^2^	23.4 ± 3.4	22.6 ± 2.9	25.9 ± 3.7	<0.001
Waist, cm	81.1 ± 9.8	78.9 ± 8.7	88 ± 9.9	<0.001
Weight, kg	67 ± 13.3	64.2 ± 11.8	75.3 ± 14	<0.001
Higher education, %	33,462 (83.79)	25,348 (84.63)	8114 (81.27)	<0.001
SBP, mmHg	109 ± 12.1	107.2 ± 11.5	114.7 ± 12	<0.001
DBP, mmHg	69.9 ± 9.4	68.6 ± 9	73.5 ± 9.5	<0.001
Fasting glucose, mg/dL	93.7 ± 10.9	91.5 ± 8	100.3 ± 14.9	<0.001
Total cholesterol, mg/dL	190.5 ± 33.1	188.6 ± 32.3	196.2 ± 34.7	<0.001
LDL-C, mg/dL	127.1 ± 32.1	124.8 ± 31.6	134.1 ± 32.7	<0.001
HDL-C, mg/dL	61.2 ± 16.6	63.9 ± 16.5	53.3 ± 14.3	<0.001
Triglycerides, mg/dL	91 (65–135)	82 (60–115)	134 (94–193)	<0.001
AST, IU/L	20 (16–24)	19 (16–23)	22 (17–28)	<0.001
ALT, IU/L	19 (13–28)	17 (13–25)	27 (17–42)	<0.001
rGTP, IU/L	20 (14–34)	18 (13–29)	31 (19–52)	<0.001
NT-proBNP, ng/L	20.77 (12.79–35.8)	22.8 (14.3–38.7)	15.5 (9.82–26.86)	<0.001
eGFR, ml/min/1.73 m^2^	104.1 ± 13.2	104.2 ± 13	103.6 ± 13.5	<0.001
Fatty liver, %	11,704 (29.3)	5896 (19.7)	5808 (58.2)	<0.001

Numbers in the table are mean (standard deviation), median (interquartile range), or percentages. ALT = alanine aminotransferase; AST = aspartate aminotransferase; BMI = body mass index; DBP = diastolic blood pressure; eGFR = estimated glomerular filtration rate; HDL-C = high-density lipoprotein cholesterol; HEPA = health enhancing physical activity; HOMA-IR = homeostasis model assessment-estimated insulin resistance; IPAQ = international physical activity questionnaire; LDL-C = low-density lipoprotein cholesterol; NT-proBNP = N-terminal pro-B-type natriuretic peptide; rGTP = γ-glutamyl transpeptidase; SBP = systolic blood pressure. High alcohol intake defined as >30 g/day for men and >20 g/day for women; higher education defined as college graduate or higher.

**Table 3:** Baseline characteristics according to sex.

	Total (*n* = 39,923)	Male (*n* = 23,009)	Female (*n* = 16,914)	*p* Value
Age	39.1 ± 7.6	39.5 ± 7.5	38.4 ± 7.7	<0.001
IPAQ %				<0.001
sedentary	19,501 (48.83)	9626 (41.83)	9875 (58.35)	
Mild	14,294 (35.79)	9373 (40.73)	4921 (29.08)	
HEPA	6001 (15.03)	3950 (17.16)	2051 (12.12)	
Unknown	141 (0.35)	64 (0.28)	77 (0.45)	
Current smoker, %	4817 (12.06)	4625 (20.1)	192 (1.13)	<0.001
Alcohol intake (g/day)	4 (1–13)	7 (3–19)	2 (0–6)	<0.001
High alcohol intake, %	3967 (10.59)	3138 (14.06)	829 (5.47)	<0.001
BMI, kg/m^2^	23.4 ± 3.4	24.7 ± 3	21.7 ± 3.1	<0.001
Waist, cm	81.1 ± 9.8	86 ± 8	74.6 ± 8.2	<0.001
Weight, kg	67 ± 13.3	74.8 ± 10.4	56.3 ± 8.7	<0.001
Higher education, %	33,462 (83.79)	20,298 (88.2)	13,164 (77.78)	<0.001
SBP, mmHg	109 ± 12.1	113.8 ± 10.9	102.6 ± 10.4	<0.001
DBP, mmHg	69.9 ± 9.4	73.2 ± 8.8	65.3 ± 8.1	<0.001
Fasting glucose, mg/dL	93.7 ± 10.9	95.7 ± 11.2	91.1 ± 9.8	<0.001
Total cholesterol, mg/dL	190.5 ± 33.1	195.8 ± 33.3	183.3 ± 31.3	<0.001
LDL-C, mg/dL	127.1 ± 32.1	135.1 ± 31.5	116.2 ± 29.7	<0.001
HDL-C, mg/dL	61.2 ± 16.6	55 ± 13.9	69.7 ± 16.3	<0.001
Triglycerides, mg/dL	91 (65–135)	112 (79–162)	71 (55–97)	<0.001
AST, IU/L	20 (16–24)	22 (18–27)	17 (15–20)	<0.001
ALT, IU/L	19 (13–28)	24 (18–35)	13 (11–18)	<0.001
rGTP, IU/L	20 (14–34)	28 (20–45)	13 (11–18)	<0.001
NT-proBNP, ng/L	20.77 (12.79–35.8)	14.6 (9.43–23.38)	32.8 (21.3–50.3)	<0.001
eGFR, ml/min/1.73 m^2^	104.1 ± 13.2	100.4 ± 12.7	109.1 ± 12.2	<0.001
HOMA IR (median (IQR))	1.38 (0.93–2.03)	1.49 (1–2.2)	1.25 (0.84–1.82)	<0.001
Fatty liver, %	11,704 (29.3)	9904 (43.0)	1800 (10.6)	<0.001

Numbers in the table are mean (standard deviation), median (interquartile range), or percentages. ALT = alanine aminotransferase; AST = aspartate aminotransferase; BMI = body mass index; DBP = diastolic blood pressure; eGFR = estimated glomerular filtration rate; HDL-C = high-density lipoprotein cholesterol; HEPA = health enhancing physical activity; HOMA-IR = homeostasis model assessment-estimated insulin resistance; IPAQ = international physical activity questionnaire; LDL-C = low-density lipoprotein cholesterol; NT-proBNP = N-terminal pro-B-type natriuretic peptide; rGTP = γ-glutamyl transpeptidase; SBP = systolic blood pressure. High alcohol intake defined as >30 g/day for men and >20 g/day for women; higher education defined as college graduate or higher.

**Table 4:** Association between fatty liver, HOMA-IR index and BMI with NT-proBNP.

	Total (*n* = 39,923)	Male (*n* = 23,009)	Female (*n* = 16,914)
Fatty liver			
No	1 (reference)	1 (reference)	1 (reference)
Yes	0.833 (0.819–0.848)	0.831 (0.814–0.848)	0.795 (0.765–0.826)
Fatty liver *			
No	1 (reference)	1 (reference)	1 (reference)
Yes	0.864 (0.849–0.880)	0.849 (0.816–0.884)	0.856 (0.839–0.874)
HOMA IR			
75% <	1 (reference)	1 (reference)	1 (reference)
75% ≥	0.859 (0.844–0.874)	0.859 (0.841–0.877)	0.844 (0.820–0.869)
BMI			
BMI < 25	1 (reference)	1 (reference)	1 (reference)
BMI ≥ 25	0.951 (0.931–0.971)	0.939 (0.916–0.962)	0.939 (0.9–0.978)

The multivariate model was adjusted for age, sex, waist circumference, systolic blood pressure, smoking status, daily alcohol consumption, estimated glomerular filtration rate, and education level. * The multivariate model was adjusted for age, sex, body mass index, systolic blood pressure, smoking status, daily alcohol consumption, estimated glomerular filtration rate, education level and homeostasis model assessment-estimated insulin resistance index.

**Table 5:** Association of NT-proBNP with fatty liver and HOMA-IR score, accounting for obesity.

	Total (*n* = 39,923)	Male (*n* = 23,009)	Female (*n* = 16,914)
Fatty liver *- high HOMA IR index **			
No-No	1 (reference)	1 (reference)	1 (reference)
No-Yes	0.888 (0.868–0.908)	0.900 (0.871–0.929)	0.869 (0.842–0.897)
Yes-No	0.856 (0.838–0.874)	0.854 (0.834–0.875)	0.825 (0.783–0.869)
Yes-Yes	0.755 (0.737–0.773)	0.752 (0.732–0.773)	0.712 (0.783–0.869)
*p* for trend	<0.001	<0.001	<0.001

* Yes→Presence of fatty liver on abdominal ultrasound. ** Yes→HOMA IR index ≥ 75%. The multivariate model was adjusted for age, sex, waist circumference, systolic blood pressure, smoking status, daily alcohol consumption, estimated glomerular filtration rate, and education level. The reference group was group of individuals both without fatty liver and high HOMA-IR index.

## Data Availability

Data sharing not applicable.
